# Obstetric Considerations in Pregnant Women with Crohn’s Disease

**DOI:** 10.3390/jcm12020684

**Published:** 2023-01-15

**Authors:** Konstantina Rosiou, Christian P. Selinger

**Affiliations:** 1 Leeds Teaching Hospital NHS Trust, Gastroenterology, Leeds LS9 7TF, UK; 2 Research Institute at St James Hospital, University of Leeds, Leeds LS9 7TF, UK

**Keywords:** inflammatory bowel disease, Crohn’s disease, pregnancy, antenatal care

## Abstract

Crohn’s disease affects many women of childbearing age. Fecundity rates are often lower than in the general population due to reduced fertility during active inflammation, effects of pelvic surgery or voluntary childlessness. Many women have concerns regarding the effects of pregnancy on their Crohn’s, any potential effect of medication on the fetus, and passing on Crohn’s disease to the offspring. International guidelines on reproduction for women with Crohn’s disease provide evidence-based advice to patients and health care professionals. There is an increasing literature on the safety of advanced medication for Crohn’s disease during pregnancy. This review article therefore focuses on obstetric considerations beyond medication safety. We provide information on fertility, factors affecting pregnancy and fetal outcomes, obstetric complications, factors influencing mode of delivery, management of intestinal stomas during pregnancy and general considerations around breast feeding.

## 1. Introduction

Crohn’s disease (CD) is a chronic inflammatory disease that can affect any part of the gastrointestinal tract with symptoms that evolve in a relapsing and remitting pattern [1]. The incidence of Inflammatory Bowel Disease (IBD) seems to be stabilizing in western countries, whereas it is rising in newly industrialised countries adopting a westernized way of living [2]. Moreover, their prevalence exceeds 0.3% in western countries making them global diseases with high burden [2]. As the onset of CD is usually between the second and fourth decade of life, many patients are of child-bearing age [2]. Understandably, patients experience anxiety regarding pregnancy in the background of IBD [3,4]. Concerns especially in terms of heredity, effects of medications and adverse pregnancy outcomes might affect patients’ choices both in family planning but also during pregnancy [4,5]. It has been shown that educational interventions can improve patient knowledge as well as anxiety and depression and quality of life for patients with IBD [6,7,8]. Management of pregnancy in women with CD can prove challenging and requires specialised care which is best done with a multidisciplinary approach.

In view of a mounting evidence on the safety of advanced therapies during pregnancy, this review article will focus on the reproductive and obstetric considerations beyond medication safety in pregnant women with CD. We will focus on the aspects that relate to women with CD, but as many studies report on IBD cohorts as a whole, some of the evidence discussed relates to ulcerative colitis (UC) or IBD in general and not just specifically CD. The medical management with standard and advanced therapies for CD during pregnancy and breast feeding has been covered very well by recent guidelines [9,10,11] and has therefore not been included in this review. We aim to provide guidance for clinicians on the other aspects of care.

## 2. Sexual Disfunction

Patients with IBD are faced with a higher prevalence of sexual disfunction, which is multifactorial in origin including the impact of psychosocial factors, disease activity, medical therapies, surgical interventions, body image perceptions and changes, hypogonadism, and pelvic floor disorders [12]. Roseira et al. have previously demonstrated that IBD patients report a lower sexual quality of life (SQoL) compared with controls [13]. Moreover, among IBD patients, SQoL was positively correlated with health-related quality of life (HRQoL) and negatively correlated with depression symptoms, and interestingly, perianal disease was associated with lower HRQoL and higher incidence of depression, but only impacted SQoL in men [13]. The results of the Danish National Birth Cohort have shown that, even though women with ulcerative colitis (UC) did not have significantly decreased sexual function, women with CD had more difficulty achieving orgasm, increased dyspareunia and deep dyspareunia compared to controls [14]. Sexual dysfunction not only affects quality of life in women with Crohn’s disease but may in itself lead to lower fecundity rates. Health care professionals providing care for women with CD should aim to explore reasons for sexual dysfunction, and where possible consider medical or surgical treatment that may decrease problems and discuss coping strategies. IBD nurses often have a different skill set to medical health care professionals and the support of IBD nurses may be especially valuable for women experiencing sexual dysfunction.

## 3. Fertility

In a UK population-based study by Ban et al., women with CD were found to have marginally lower fertility rates compared to controls [15]. Importantly, fertility rate was significantly reduced in the 9-month period following a flare of the disease [15]. Disease optimization may therefore help conception. In addition, as disease activity during pregnancy is determined by disease activity during conception, optimal control of CD prior to conception will not only help with getting pregnant but also ensure a better disease course during pregnancy. The impact of surgical interventions other than ileal pouch-anal anastomosis (IPAA) on fertility is largely unknown. In a meta-analysis by Lee et al. the authors concluded that the effect of surgical interventions is uncertain [16]. It is also uncertain if there are any differences in infertility among those undergoing open versus laparoscopic procedures, with the exception of IPAA as for those procedures a laparoscopic approach is associated with better fertility rates [16]. While previous surgery was associated with higher risk of miscarriage and use of assisted reproductive technology (ART), the findings were based on low-quality evidence [16]. Studies have shown that patients with CD had in vitro fertilization (IVF) success rates comparable to the general infertile population [17,18]. However, surgery for CD before ART treatment was found to significantly reduce the chance of live birth for each embryo transfer in a Danish nation-wide cohort study. [19] Unfortunately, many IBD health care professionals have insufficient knowledge about ART and are hesitant to initiate referrals for ART. We recommend that women with additional risk factors for infertility should be considered for ART referral early if attempts at conception are unsuccessful.

## 4. Influence of Disease Behaviour during Conception on Disease Course during Pregnancy and Pregnancy Outcomes

Patients with quiescent CD at conception have a comparable risk of flare during the next 9 months as non-pregnant patients. The prospective multicentre study by the Epidemiology Committee (EpiCom) of the European Crohn’s and Colitis Organisation (ECCO) did not demonstrate any statistically significant difference in disease course during pregnancy between pregnant and non-pregnant women with CD [20]. On the other hand, periconception disease activity has been related with disease activity during pregnancy. A meta-analysis by Abhyankar et al. included 14 studies in total and six studies on CD with 590 patients [21]. Patients with active disease at conception were more likely to have active disease during pregnancy than those who conceive when in remission [21]. While the authors conclude that studies used in the meta-analysis had high risk of bias, similar findings were observed in subsequent prospective study by de Lima-Karagiannis et al. [22] and by Rottenstreich et al. where, in multivariate analysis, active disease at conception and history of disease flare at previous pregnancy were the only independent factors of disease relapse [23]. While active disease during pregnancy has consistently been associated with poor outcomes, active IBD during the periconception period has now also been shown to lead to adverse pregnancy outcomes. A recent meta-analysis by Kim et al. suggests that active IBD during periconception and pregnancy is associated with an increased risk of adverse pregnancy outcomes involving higher risk of preterm birth, small for gestational age (SGA) and spontaneous abortion for patients with CD [24]. It is therefore vitally important to aim to achieve remission prior to conception, as also recommended by the current ECCO guidelines [9]. Health care providers should be aware that considerable proportions of pregnancy are unplanned and may therefore happen at times of less than ideal control of IBD. Efforts should be made to assess disease activity and optimize disease control when women with active IBD become pregnant.

## 5. Risk of Adverse Neonatal and Maternal Outcomes

Achieving good pregnancy outcomes for mother and fetus are the key aims of IBD antenatal care. Health care professionals need to be therefore aware of factors associated with poor outcomes to detect problems early and try and mitigate them. Population studies have previously demonstrated the importance of adequate gestational weight gain (GWG) and the association between inadequate GWG and preterm birth or small for gestational age (SGA) births in the general population [25,26]. The association of low GWG has also been investigated in women with IBD. Oron et al. demonstrated that in patients with both CD and UC, weight gain of less than 12 kg during pregnancy was significantly associated with an adverse pregnancy outcome including pre-term delivery, SGA and admission to neonatal intensive care [27]. The results of the Norwegian Mother and Child Cohort study (MoBa) also showed that women with CD were more frequently exposed to inadequate GWG compared with non-IBD mothers (34.3% vs. 19.4%) [28]. Moreover, women with IBD and inadequate GWG had a 2-fold risk for SGA births compared to women with inadequate GWG without IBD and even more interestingly, patients with CD and inadequate GWG had a several-fold increased risk for SGA compared to women with IBD with normal GWG [28]. Active IBD was associated with reduced GWG (<13 kg compared with >17.5 kg), highlighting the need for optimal disease control [28]. Furthermore, an increased risk of intrauterine growth restriction and a trend for SGA were demonstrated in CD, and flares of IBD during pregnancy increased the risk of inadequate GWG [28].

The causes of inadequate GWG have not been fully elucidated so far. There is sufficient evidence that active inflammation is associated with GWG [28]. In addition, patients with CD are prone to malnutrition and this can be present pre-conception but also during the early stages of pregnancy when placental development happens rapidly which could contribute to low GWG. Malnourishment can be related to inadequate dietary intake, malabsorption, and active disease. Guidelines advise that patients with IBD should be checked for deficiencies on a regular basis and specific deficits should be appropriately corrected. Common deficiencies include iron, folic acid, calcium, vitamins, protein, fat, and zinc [29]. A previous analysis of the Norwegian Mother and Child Cohort showed that IBD mothers with high adherence to a dietary pattern characterized by high consumption of lean fish, fish products, potatoes, rice porridge, cooked vegetables, and gravy had lower risk of SGA compared to IBD and non-IBD mothers with low adherence, the difference however was only found to be significant for UC and not CD patients [30]. A further MoBa-IBD study focused on the impact of intake of dairy protein during pregnancy and demonstrated that low and middle proportion of protein from dairy sources (PPDS) was a fourfold stronger predictor for inadequate GWG in CD, compared to non-IBD mothers [31].

In addition to GWG other risk factors associated with adverse neonatal and maternal outcomes in IBD pregnancies have been investigated by several studies. Two meta-analyses by Cornish et al. and O’Toole et al. including 1952 and 5449 patients with CD, respectively, have demonstrated an increased risk for preterm delivery, stillbirth, SGA, and low birth weight (LBW) amongst patients with IBD compared to controls [32,33]. In our own cohort of patients, we found no associations with increased risk of preterm birth, LBW or SGA [34]. IBD patients were reviewed in combined IBD antenatal clinics and were monitored closely with additional foetal growth scans, thus providing a greater opportunity to identify growth retardation or risk factors for preterm delivery which might have potentially resulted in the improved results [34].

Patients with IBD are also at higher risk of developing gestational diabetes during pregnancy based on a meta-analysis by Tandon et al. that included 53 studies, enrolling 7917 pregnancies with IBD in total [35]. Whether this relates to or can happen regardless of the use of corticosteroids remains controversial, and while Tandon et al. found no association between gestational diabetes and steroid exposure, a Canadian study found a clear association between steroid exposure and gestational diabetes [36]. Efforts to minimize corticosteroid exposure during pregnancy are welcome but should be weighed against the risk of active disease. Reassuringly, the incidence of placental diseases was low for IBD patients reaching 2.0% for pre-eclampsia, 3.3% for placental abruption, 0.5% for placenta previa and 0.3% for chorioamnionitis, and patients with IBD were more likely to experience preterm prelabour rupture of membranes, but not early loss of pregnancy [35]. Of note, Boyd et al. in a study including 278 patients with CD, demonstrated that even though the overall pre-eclampsia rate in women with IBD did not differ significantly from that in women without IBD, rates of severe pre-eclampsia were more than 2-fold greater in the IBD population [37]. A Danish cohort study by de Silva et al. showed that women with CD had increased risk of ectopic pregnancy compared to women without IBD whereas the risk was not higher in UC patients [38]. Previous surgery did numerically increase the risk of ectopic pregnancy, but the increase was not found to be statistically significant [38].

Disease flares during pregnancy are known to increase the risk of adverse pregnancy outcomes [9]. In a Swedish cohort study, having a flare further increased the risk of preterm birth, stillbirth, SGA, and low Apgar score in patients with CD [39]. Bush et al. had similarly previously demonstrated that flares during pregnancy are associated with an increased risk for preterm delivery and foetal low body weight, [40] and in a Korean cohort study including 589 patients with CD, patients with moderate to severe disease activity had lower rates of live birth compared to controls and increased rates of spontaneous abortion [41].

Venous thromboembolism (VTE) poses a serious risk to the expectant mother and thereby indirectly to the fetus. A UK population-based study has demonstrated that women with IBD had an increased risk for VTE both in the antepartum and in the 12-week postpartum period compared with women without IBD (50). These findings are supported by a Danish population-based study showing a relative risk (RR) of 1.72 (95% CI, 1.22–2.43) during pregnancy and RR of 2.10 (95% CI, 1.33–3.30) in the post-partum period [42] and a recent systematic review meta-analysis by Kim et al. [43] Caesarean section (CS) was found to be independently associated with an increased risk of VTE in a US study by Nguyen et al. [44] Guidance from the Canadian Association of Gastroenterology suggests that women with IBD who have CS should receive anticoagulant thromboprophylaxis during the hospitalized period unless postpartum haemorrhage has occurred. Moreover, if there is a history of VTE, prophylaxis for up to 6 weeks after delivery is advised [45]. In addition, British Royal College of Gynecologists and Obstetricians guidelines recommend VTE prophylaxis for pregnant women with IBD who experience active disease during the third trimester of pregnancy [46].

**Figure 1:**
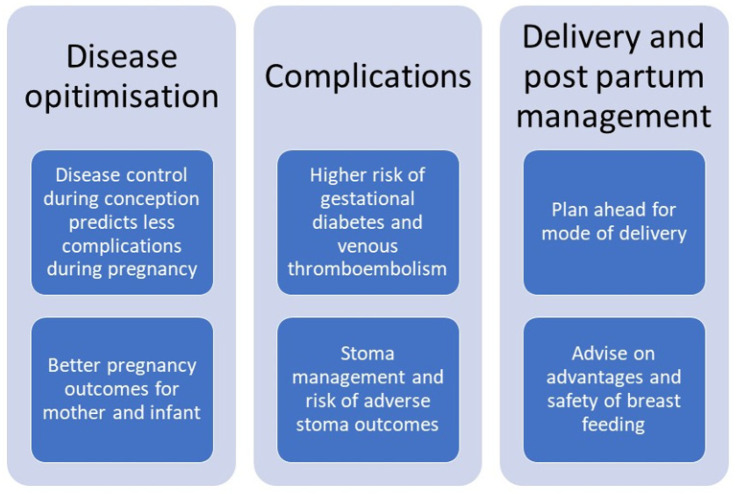
Considerations for antenatal care in women with IBD.

Overall, active disease is the main predictor of adverse pregnancy outcomes which once again highlights the need to achieve remission both during the preconception period and during pregnancy (Figure 1). Health care professionals should make every effort to achieve disease remission and counsel patients on the importance of achieving and maintaining remission. In lay terms “a healthy mother is important for having a healthy baby”.

## 6. Mode of Delivery

Many factors influence the preferred mode of delivery in general. Medical factors including those affecting maternal or foetal health, factors related to the progression of labour and potential long-term effects related to mode of delivery may lead to a clear preference for a mode of delivery, but patient choice plays an increasing role in choosing the mode of delivery. Some patients perceive CS to be a “safe choice for the mother” [47] but it is important to remember that CS is major open surgery with associated risks. Delivery by CS has implications on future mode of delivery. Vaginal delivery in subsequent pregnancies is possible but may pose a slightly increased risk of uterine rupture, but there is also risk in performing multiple CS during a women’s life [48]. VTE risk is significantly higher in women undergoing CS [43]. CS is unfortunately associated with small but significant increase in still birth in subsequent pregnancies [49]. In addition, infants borne by CS experience differences in their microbiome and are, for example, at higher risk of atopic diseases [50]. Vaginal delivery carries the risk of significant tears [51] and pelvic floor problems [52]. It is therefore important to take these aspects into account when deciding on the optimal mode of delivery.

Several studies have demonstrated increased rates of caesarean section (CS) among patients with IBD [27,32,34,53,54,55] however, the reasons for this increase are still not fully elucidated [56]. We have previously shown that elective CS in IBD patients occurred in 40% for indications related to their IBD. [34] The meta-analysis by Cornish et al. in 2007 showed that the incidence of caesarean section in patients with CD was significantly higher compared to controls (OR 1.65; 95% CI 1.19 to 2.29; *p* = 0.003), interestingly such a correlation was not found in patients with UC [32]. On the other hand, the systematic review and meta-analysis by Tandon et al. demonstrated increased rates of CS for IBD patients; however, the difference was not statistically significant for patients with CD [35]. Among patients with CD, previous intestinal or perianal surgery and active perianal disease were associated with an increased risk of CS [35]. Data from our cohort of patients also demonstrated that UC rather than CD was associated with an increased risk of CS compared with non-IBD patients, whereas CD demonstrated an increased risk of elective CS, but not all CS including emergency and elective CS [34,52].

The impact of perianal CD on delivery mode and potential complications have been previously investigated. In this systematic review by Foulon et al., seven studies with 544 CD patients and follow-up ranging from 2 months to 5.2 years were included [57]. In patients with inactive perianal disease, the study did not demonstrate any increased risk of new perianal disease or recurrence of perianal disease when comparing vaginal delivery to CS. However, for patients with active disease, worsening of symptoms was noted in two-thirds of cases [57]. There are particular concerns regarding 3rd and 4th degree tears in patients with active perianal CD as it is unlikely that good healing can be achieved in infected and inflamed perineal tissues in these cases. Active perianal CD is therefore a mandatory indication for elective CS [9]. In patients with no history of prior perianal CD episiotomy, perianal tears, and instrumental delivery did not influence the incidence of perianal CD [57]. There is therefore no need to avoid vaginal deliveries to prevent the development of perianal CD.

The risk of significant perineal tears during vaginal deliveries has been investigated by few studies only. A retrospective study by Hatch et al. showed that the rates of 4th degree perineal lacerations were similar between patients with CD without perianal involvement compared to controls, but these rates increased significantly in patients with perianal disease (12.3%, *p* < 0.001) [58]. Reassuringly, data from our cohort of patients showed that previous perianal disease was not associated with significant perineal trauma [52]. The risk of 3rd or 4th degree tears was not increased in patients with IBD compared to general population controls [52].

Very few studies have investigated the impact of vaginal delivery on faecal incontinence. In a study by Ong et al., among 129 patients with CD, 21 patients reported faecal incontinence and 8 of them dated their symptoms back to the time of vaginal delivery. While the difference was significantly higher compared to non-IBD controls [59], significant concerns over selection and recall bias limit the conclusions [59]. On the other hand, in a small study of 57 women comparing patients with UC and CD to non-IBD controls, patients with IBD in remission did not have higher incidence of post-partum anal incontinence [60]. Moreover, a large UK study with more than 1500 patients with CD, no association was found between faecal incontinence and vaginal delivery in multivariate analysis [61]. In keeping with these findings, data from our cohort of IBD patients support that perineal trauma is uncommon both in IBD and non-IBD patients. Four IBD patients experienced clinically significant tears, but none had pelvic floor dysfunction or incontinence at follow-up, and only one IBD patient who had a clinically non-significant second-degree perineal tear reported incontinence a year after vaginal delivery [52].

Data on the impact of the mode of delivery in the development of IBD in the offspring have been conflicting. In a systematic review and meta-analysis by Bruce et al., no significant difference in the risk of IBD in offspring was observed when comparing children delivered by CS with those born vaginally [62]. Similar findings were found in a population study by Bernstein et al. [63] On the other hand, a population-based Danish study showed that being born by CS can increase the risk of chronic inflammatory diseases including IBD [64] and a recent population-based study from Sweden suggests that CS increases the risk of CD later in life [65].

Current guidelines suggest that the mode of delivery for patients with CD should primarily be guided by obstetric considerations [9,10,11,66]. An exception lies with patients with active perianal disease or previous rectovaginal fistula where CS is the preferred mode of delivery [9]. Discussion about the mode of delivery should begin early in pregnancy and involve the gastroenterologist, obstetrician, the patient and in selected cases a dedicated IBD surgeon.

## 7. Management of the Pregnant Patient after Stoma Surgery for IBD

Special consideration should be given in the management of pregnant patients with an existing ileostomy or colostomy. These patients have often undergone multiple abdominal surgeries and therefore often have complex medical and surgical problems. The largest study to date is a retrospective UK audit of 78 pregnancies in women with IBD and ileostomies or colostomies [67]. The study demonstrated that pregnancy for women who previously had stoma formation is associated with higher rates of caesarean section and stoma complications [67]. Significant stoma related complications occurred in approximately one quarter of pregnancies and included stoma prolapse, parastomal hernias and small bowel obstructions [67]. Interestingly, stoma complications were observed only in patients with an ileostomy and not a colostomy and occasionally surgery was required to manage these complications [67]. In general, pregnancy in patients with a stoma leads to good outcome but higher adverse events rate than otherwise expected reflect the complex background situation for many of these patients. They need to be counselled early about the associated risks and require close follow-up to ensure that complications are detected and managed timely.

## 8. Post-Partum Management and Breastfeeding

The main considerations in the post-partum period include maintaining control of IBD, mental health monitoring, and considerations around infant feeding. The ECCO EpiCom study that included 209 pregnant IBD patients found no increased risk of CD relapse postpartum, in contrast to UC where an increased risk of flare both during pregnancy and postpartum was observed [20]. In the study by Yu et al. that included women with both CD (47%) and UC (53%), one third of patients experienced a postpartum flare within the year after delivery [68]. Development of a postpartum flare was predicted by disease activity during the third trimester of pregnancy, therapy de-escalation during or after pregnancy, but was not related to disease type, duration of disease, or mode of childbirth [68]. Similarly, in a systematic review and meta-analysis, it was shown that active disease at conception or during pregnancy, biologic discontinuation in the third trimester and therapy de-escalation after delivery were associated with postpartum disease activity [69]. Moreover, patients with stricturing and penetrating CD had higher odds of postpartum active disease [69]. Finally, this Danish study of patients with Crohn’s disease demonstrated that smoking and non-adherence to medications were associated with increased risk of relapse of disease in the postpartum period [70]. Avoidance of post-partum flares or at least rapid treatment of post-partum flares is required to offer women the best conditions when looking after a newborn, and health care professionals should therefore aim to optimize care in view to minimize this risk.

Vigod et al. have shown that patients with CD are at increased risk for new-onset mental health illness in the post-partum period compared to controls, and the risk was specifically elevated for mood or anxiety disorder and alcohol or substance use disorders [71]. Healthcare providers should therefore aim to achieve early recognition and treatment of these conditions. As the main contact with women in the post-partum period is with midwives and health visitors, these should be made aware of the increased risk in women with IBD.

Several health benefits can be derived from breastfeeding. Aside from being natural nutrition, breast milk contains protective factors against infectious diseases but may also influence the development of the immune system [72]. Two previous meta-analysis have suggested that breastfeeding may be protective against the development of paediatric onset IBD however, the quality of existing data was considered poor in both studies [73,74]. There is also lack of data on the specific and high-risk population of children born by mothers with IBD as genetics will play a stronger factor than in the general population. Data on the impact of breastfeeding on IBD activity are inconsistent, coming from small retrospective studies that have reported anything from protective effect, [70] increased risk of relapse on breastfeeding women with CD [although the association was not significant when corrected for medication cessation] [75], or no impact of breastfeeding on disease activity [53,76]. Latest ECCO guidelines therefore conclude that breastfeeding does not seem to impact on disease activity in mother’s with IBD [9]. Women with IBD should be made aware of the positive effects of breast feeding and this needs to be balanced against any potential (usually small) risk that may arise from medication exposure.

## 9. Conclusions

Pregnancy is a very delicate period in a woman’s life and can be associated with significant challenges, especially for patients suffering from chronic diseases who can experience increased levels of anxiety related to the impact of their disease and medications on pregnancy and the offspring. Fortunately, most patients will have an uncomplicated pregnancy and the absolute risk of complications remains low. Active CD is the main risk for adverse pregnancy outcomes and every effort should be put on maintaining remission of the disease. As fertility can be impaired by active inflammation or after deep pelvic surgery, referral for advanced reproductive techniques should be considered early when patients struggle to conceive. To avoid adverse pregnancy and fetal outcomes, intestinal inflammation should be minimised and nutrition optimised. Clinicians should be aware of the increased risk of gestational diabetes and VTE. Most women with CD should be able to have a vaginal delivery. Complication rates are high in women with intestinal stomas during pregnancy and close follow-up is required. Breast feeding provides optimal nutrition for the infant and should be encouraged. Women with CD should have preconception counselling and close monitoring during the antenatal and post-partum period to detect and manage complications in a timely manner. This care is best provided in a multidisciplinary setting through dedicated clinics.

## Data Availability

Not applicable.
